# PET imaging of cyclooxygenase-2 (COX-2) in a pre-clinical colorectal cancer model

**DOI:** 10.1186/s13550-016-0192-9

**Published:** 2016-04-26

**Authors:** Ole Tietz, Melinda Wuest, Alison Marshall, Darryl Glubrecht, Ingrit Hamann, Monica Wang, Cody Bergman, Jenilee D. Way, Frank Wuest

**Affiliations:** Department of Oncology, University of Alberta, 11560- University Avenue, Edmonton, AB T6G 1Z2 Canada; Faculty of Pharmacy and Pharmaceutical Sciences, University of Alberta, Edmonton, Canada

**Keywords:** Cyclooxygenase-2 (COX-2), Positron emission tomography (PET), Colorectal cancer, COX-2 inhibitor

## Abstract

**Background:**

Cyclooxygenase-2 (COX-2) is the inducible isoform of the cyclooxygenase enzyme family. COX-2 is involved in tumor development and progression, and frequent overexpression of COX-2 in a variety of human cancers has made COX-2 an important drug target for cancer treatment. Non-invasive imaging of COX-2 expression in cancer would be useful for assessing COX-2-mediated effects on chemoprevention and radiosensitization using COX-2 inhibitors as an emerging class of anti-cancer drugs, especially for colorectal cancer. Herein, we describe the radiopharmacological analysis of [^18^F]Pyricoxib, a novel radiolabeled COX-2 inhibitor, for specific PET imaging of COX-2 in colorectal cancer.

**Methods:**

Uptake of [^18^F]Pyricoxib was assessed in human colorectal cancer cell lines HCA-7 (COX-2 positive) and HCT-116 (COX-2 negative). Standard COX-2 inhibitors were used to test for specificity of [^18^F]Pyricoxib for COX-2 binding in vitro and in vivo. PET imaging, biodistribution, and radiometabolite analyses were included into radiopharmacological evaluation of [^18^F]Pyricoxib.

**Results:**

Radiotracer uptake in COX-2 positive HCA-7 cells was significantly higher than in COX-2 negative HCT-116 cells (*P* < 0.05). COX-2 inhibitors, celecoxib, rofecoxib, and SC58125, blocked uptake of [^18^F]Pyricoxib in HCA-7 cells in a concentration-dependent manner. The radiotracer was slowly metabolized in mice, with approximately 60 % of intact compound after 2 h post-injection. Selective COX-2-mediated tumor uptake of [^18^F]Pyricoxib in HCA-7 xenografts was confirmed in vivo. Celecoxib (100 mg/kg) selectively blocked tumor uptake by 16 % (PET image analysis; *P* < 0.05) and by 51 % (biodistribution studies; *P* < 0.01).

**Conclusions:**

The novel PET radiotracer [^18^F]Pyricoxib displays a promising radiopharmacological profile to study COX-2 expression in cancer in vivo.

**Electronic supplementary material:**

The online version of this article (doi:10.1186/s13550-016-0192-9) contains supplementary material, which is available to authorized users.

## Background

Cyclooxygenases (COXs) are responsible for the complex conversion of arachidonic acid into prostaglandins, which exert as autocrine or paracrine messengers a wide range of physiological functions mediated through binding to prostaglandin E (EP) G-protein-coupled receptors (EP1-EP4) [1]. The COX enzyme family consists of two distinct isoforms; COX-1, which is a constitutively expressed enzyme; and COX-2, which is the inducible form of the enzyme. COX-1 functions as a housekeeping enzyme and is expressed in most tissues types. The enzyme is responsible for maintaining homeostasis (gastric and renal integrity) and normal production of eicosanes [1]. COX-2 is only expressed in response to inflammatory stimuli and virtually absent in most resting tissues [2]. COX-2 expression is usually significantly upregulated under acute and chronic inflammatory conditions [3], as well as in neurodegenerative diseases like Parkinson’s and Alzheimer’s [4] and a variety of cancers [5].

COX-2 has become an extensively studied drug target, and numerous selective COX-2 inhibitors (coxibs) have been developed [6]. Various selective COX-2 inhibitors were in widespread clinical use from when they first gained FDA approval in 2000. However, most coxibs were withdrawn from the market in 2005 following concerns over their cardiac safety profiles [7]. Despite its evident involvement in a variety of disease conditions, the multiple pathogenic and non-pathogenic roles of COX-2 in human physiology have not been fully explored yet. A recent shift in COX paradigm suggests that balance between COX-1 and COX-2 expression is of crucial importance. Recent studies on biochemical mechanisms that underline the cardiac toxicity of coxibs support this theory [8, 9].

Elevated COX-2 expression was also demonstrated in many human cancers such as colorectal, gastric, and breast cancer [10–13]. Although several aspects of the molecular mechanisms underlying COX-2 expression in cancer and inflammatory lesions have been elucidated [14], there are discrepancies between the potent anti-cancer effects of several COX-2 inhibitors in pre-clinical studies and their failure in the majority of clinical trials [15]. The development of techniques for non-invasive monitoring of COX-2 functional expression would greatly facilitate efforts to understand the COX-2 pharmacology in a living organism.

To date, an exact assessment of COX-2 expression can only be achieved by laborious analyses ex vivo. Ex vivo analysis of COX-2 is not particularly accurate since COX-2 mRNA and protein are not stable outside the body and degrade rapidly [16]. Nuclear molecular imaging techniques such as positron emission tomography (PET) and single-photon emission computed tomography (SPECT) would provide unique opportunities to collect data on COX-2 expression levels in vivo during disease development, its progression, and the involvement of COX-2 in various diseases. Over the past decade, more than two dozen of PET and SPECT radiotracers for COX-2 imaging have been developed. A comprehensive overview of the advances in the field and the challenges surrounding the identification of a suitable radiotracer for COX-2 imaging has been the subject of several recent reviews [17–19]. However, despite the large number of structurally diverse radiolabeled COX-2 inhibitors, most of them based on the celecoxib backbone [20, 21], none was suitable for molecular imaging of COX-2 in pre-clinical models of cancer due to lack of sufficient uptake levels in vivo. A recent attempt from our research team transforming a successful in vitro fluorescence-labeled celecoxib derivative into a ^18^F-labeled radiotracer also failed because no sufficient trapping in COX-2 expressing tumors was detected [22]. Based on that, the goal of the present study was to evaluate an alternative COX-2 radiotracer based on a pyrimidine scaffold [23] for the first time in vivo. We wanted to analyze if [^18^F]Pyricoxib, which radiosynthesis we had developed recently, would be a better PET imaging probe for assessment of functional expression of COX-2 in a pre-clinical human colorectal cancer model in vitro and in vivo.

## Methods

### Radiochemistry

Radiosynthesis of [^18^F]Pyricoxib was performed as recently described [20]. Details on the radiosynthesis and the formulation of [^18^F]Pyricoxib for in vitro and in vivo studies are described in detail in the Additional file 1.

### Cell uptake studies

In vitro evaluation of [^18^F]Pyricoxib was performed with cell lines HCA-7 (human colon adenocarcinoma; colony 29, ECACC 2091238) and HCT-116 (human colorectal carcinoma; ATCC CCL-247). Cellular uptake experiments using [^18^F]Pyricoxib (300 kBq/mL; specific activity >40 GBq/μmol) were performed in triplicates in Krebs buffer at 37 °C with 5-, 10-, 15-, 30-, 60-, 90-, and 120-min incubation time. For blocking studies, cells were pre-incubated for 30 min with 10 and 100 μM of Pyricoxib, celecoxib, rofecoxib, or SC58125 prior to the addition of [^18^F]Pyricoxib.

Blocking experiments were performed at 60 min. Radiotracer uptake was stopped by the addition of 1 mL of ice-cold PBS. Then, cells were washed two times with PBS and lysed in 0.4 mL of radioimmunoprecipitation assay buffer (RIPA buffer). Radioactivity of cell lysates was determined with a WIZARD2 Automatic gamma counter (Perkin Elmer; Waltham, MA, USA). Total protein concentration in the samples was determined by the bicinchoninic acid method (BCA; Pierce, Thermo Scientific 23227) using bovine serum albumin (800, 600, 400, 300, 200, 100, 50 μg/mL, blank) as protein standard. Cell uptake data are expressed as percent of measured radioactivity per 1 mg protein (%radioactivity/mg protein). Further information on cell culture protocols can be found in the Additional file 1.

### In vivo tumor model

All animal experiments were carried out in accordance with the guidelines of the Canadian Council on Animal Care (CCAC) and approved by the local animal care committee (Cross Cancer Institute, University of Alberta).

Positron emission tomography (PET) and biodistribution experiments were carried out in HCA-7 and HCT-116 tumor-bearing NIH-III nude mice (Charles River Laboratories, Quebec, Canada). Female NIH-III nude mice were housed under standard conditions with free access to standard food and tap water. HCA-7 and HCT-116 cells (5 × 10^6^ cells in 100 μL of PBS) were injected into the upper left flank of female NIH-III nude mice (20–24 g). After 14 to 21 days post-inoculation, HCA-7 and HCT-116 tumors reached sizes of approximately 0.6–0.9 cm^3^ (0.78 ± 0.15 g, as determined during biodistribution experiments) which were suitable for all in vivo experiments.

### Radiometabolite analysis

The radiotracer [^18^F]Pyricoxib (10 MBq) was injected intravenously into female NIH-III nude mice under isoflurane anesthesia. Blood samples from the tail vein (20–40 μL) were collected at 5, 30, 60, and 120 min p.i. Plasma was separated by centrifugation (5 min, 13,000×*g*) followed by plasma protein precipitation using ice-cold methanol (two parts per one part plasma) and centrifugation (5 min, 13,000×*g*). Supernatants were analyzed by radio thin-layer chromatography (radio-TLC). TLCs were developed in 1 % MeOH/CH_2_Cl_2_ and analyzed using a BAS-5000 reader. [^18^F]Pyricoxib had an *R*_*f*_ of 0.45 to 0.50 in this solvent system.

### Biodistribution studies in mice

NIH-III mice (body weight 21 ± 2 g) bearing subcutaneous HCA-7 tumors were intravenously injected with 3–7 MBq of [^18^F]Pyricoxib in 200 μL of solvent (10 % EtOH/H_2_O). A second group of NIH-III mice (body weight 21 ± 2 g) bearing HCA-7 tumors were treated with 2 mg of celecoxib administered via intraperitoneal injection in 100 % DMSO 60 min prior to intravenous injection of [^18^F]Pyricoxib (3–7 MBq) in 200 μL of solvent (10 % EtOH/H_2_O). Animals were sacrificed at 60 min p.i. The organs and tissues of interest were rapidly excised, weighed, and the radioactivity was determined using the automatic gamma counter (see above). Radioactivity in the selected tissues and organs was calculated as percent injected dose per gram tissue (%ID/g). Data were analyzed as means ± standard deviation (mean ± SD) for *n* = 4 animals.

### Pre-clinical PET imaging

General anesthesia of HCA-7 tumor-bearing mice was induced with inhalation of isoflurane in 40 % oxygen/60 % nitrogen (gas flow = 1 mL/min), and mice were subsequently fixed in prone position. The body temperature was kept constant at 37 °C for the entire experiment. For PET experiments, 3–8 MBq of [^18^F]Pyricoxib in 150 μL of solution (formulation see Additional file 1) was administered intravenously as a bolus injection into the tail vein. PET data was collected dynamically over 60 min for up to 4 h using an Inveon^®^ PET/CT scanner (Siemens Preclinical Solutions, Knoxville, TN, USA). For the initial 4-h dynamic PET experiments, 3.5 % HSA was added to the final injection solution, while all other experiments were done without addition of carrier protein HSA. Mean standardized uptake values [SUV_mean_ = (activity/mL tissue)/(injected activity/body weight), mL/g] were calculated for each region of interest (ROI) with a threshold defined at 50 % of radioactivity uptake. The time-activity curves (TACs) were generated from dynamic PET scans. All semi-quantified PET data are presented as means ± SEM. In the blocking experiments, COX-2 inhibitor celecoxib (2 mg per animal) in 100 μL DMSO was injected intraperitoneally 60 min prior to radiotracer administration. Detailed information on PET acquisition and data analysis can be found in the Additional file 1.

### Protein analysis

Standard Western blotting methods were used to determine COX-2 and COX-1 protein content. Detailed description can be found in the Additional file 1.

### Immunohistochemistry for detection of COX-2 and CD68

Triplicates of HCA-7 and HCT116 tumors were excised from euthanized mice, fixed in neutral buffered 10 % formalin overnight and embedded in paraffin. The sections of 4-μm thickness were dried in an oven at 60 °C for 1 h. The sections were rehydrated by placing the slides in three changes of xylene for 10 min each, then graded ethanol from 100 to 50 %, followed by water and Tris-buffered saline. Slides were microwaved in a pressure cooker for 6 min in citraconic anhydride (0.05 % in water, pH 7.4) for antigen retrieval.

Slides were blocked with 0.5 % fish gelatine in Tris-buffered saline with 0.05 % Tween-20 (TBST) and incubated with mouse monoclonal anti-CD68 antibody (clone KPI, sc-20060, Santa Cruz Biotechnology, 1:300) or goat polyclonal anti-COX-2 (clone M-19, sc-1747, Santa Cruz Biotechnology, 1:3000) in a humidity chamber overnight at 4 °C. After incubation in 3 % H_2_O_2_ in water for 15 min, samples for detecting CD68 were incubated with DakoCytomation Envision + anti-mouse HRP-labelled polymer (DakoCytomation, Glostrup, Denmark) for 1 h, while samples for detecting COX-2 were treated with Goat Probe for 15 min, followed by Goat-on-Rodent HRP-polymer (Biocare Medical, Concord, USA) for another 15 min. Slides were developed, using Dako Liquid DAB+ Substrate Chromagen System plus 1 % copper sulfate and counterstained with hematoxylin. Slides were dehydrated by reversing rehydration procedure and cover slipped.

### Statistical analysis

All in vitro data are expressed as means ± SEM, all in vivo data as means ± SD. Graphs were constructed using GraphPad Prism 4.0 (GraphPad Software). Where applicable, statistical differences were tested by unpaired Student’s *t* test and were considered significant for *P* < 0.05.

## Results

### Chemistry and radiochemistry

**Fig. 1:**
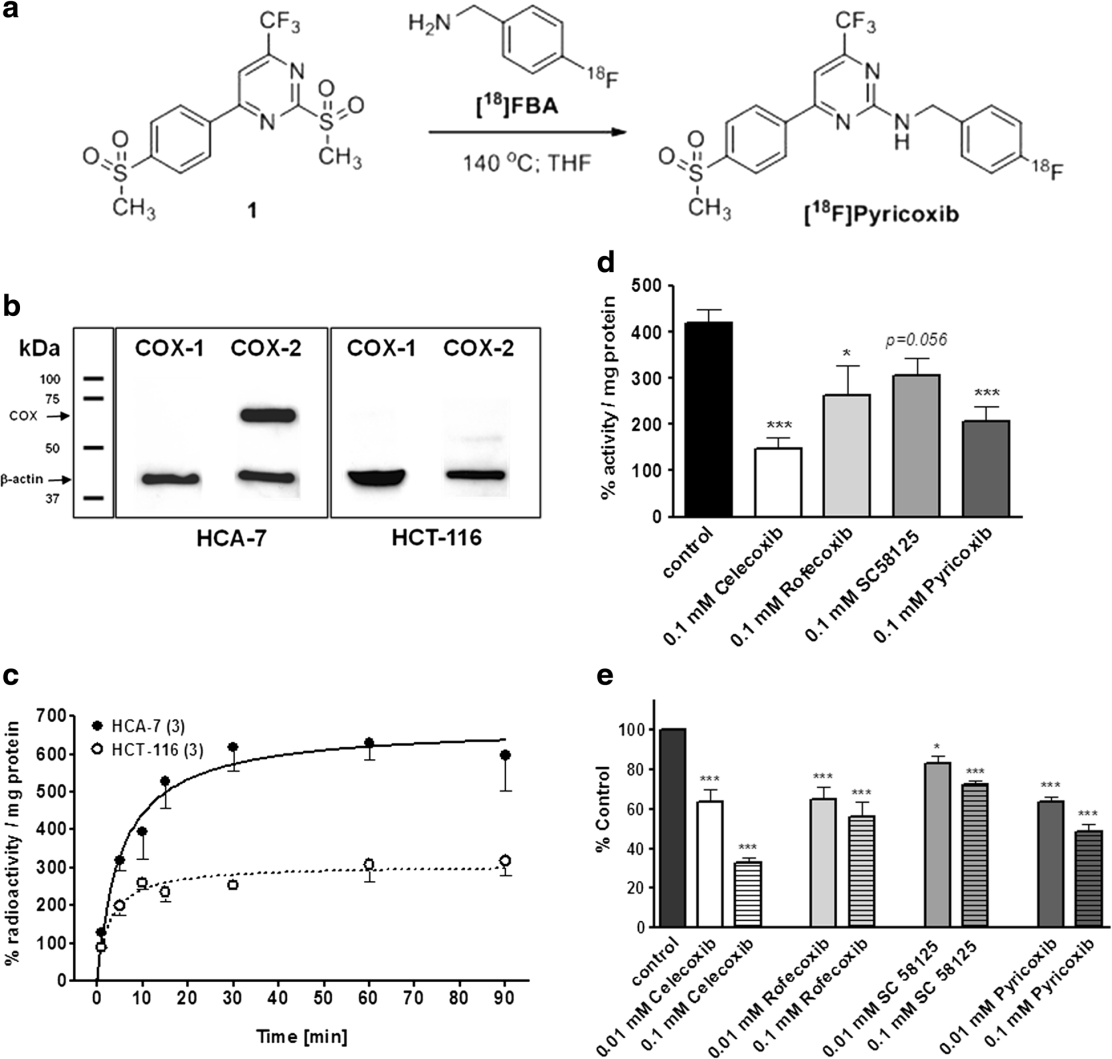
**a** Radiosynthesis of [^18^F]Pyricoxib utilizing building block 4-[^18^F]fluoro-benzylamine ([^18^F]FBA). **b** Western blot analysis of COX-1 and COX-2 in cell lysates of HCA-7 and HCT-116 cell lines. **c** Uptake of [^18^F]Pyricoxib into HCA-7 and HCT-116 cells over 90 min. Data are normalized as %radioactivity per mg protein and shown as mean ± SEM from *n* = 3 experiments. **d** Blocking of [^18^F]Pyricoxib uptake (60-min incubation time) into HCA-7 cells using high concentrations (0.1 mM) of different selective COX-2 inhibitors. Data are normalized as % radioactivity per mg protein and shown as mean ± SEM from nine data points out of three experiments. **e** Concentration dependent inhibition of [^18^F]Pyricoxib uptake (60-min incubation time) into HCA-7 cells. Data are normalized as % maximum uptake of [^18^F]Pyricoxib and analyzed as mean ± SEM from nine data points out of three experiments. *P < 0.05; ***P < 0.001

Synthesis of Pyricoxib-labeling precursor (*N*-(4-fluorobenzyl)-4-[4-(methylsulfonyl)-phenyl]-6-(trifluoro-methyl)pyrimidin-2-amine) **1** and [^18^F]Pyricoxib have previously been described by Tietz et al. [24, 25]. Pyricoxib was evaluated for its COX-2 inhibitory potency and selectivity profile in an in vitro inhibition assay. Pyricoxib displayed excellent COX-2 inhibitory potency (IC_50_ 7 nM) which was higher than that of celecoxib (IC_50_ 40 nM). Pyricoxib did not show COX-1 inhibition in the concentration range tested [24]. Radiosynthesis of [^18^F]Pyricoxib based on the reaction of bis-methylsulfone precursor **1** with 4-[^18^F]fluorobenzylamine ([^18^F]FBA) was accomplished within 95 min including HPLC purification in radiochemical yields of 27 ± 11 % (Fig. 1a).

### Cell uptake studies

Human colorectal cancer cell lines HCA-7 (COX-2 positive) and HCT-116 (COX-2 negative) were used to study the uptake of [^18^F]Pyricoxib in vitro. High baseline expression of COX-2 in HCA-7 cells is well documented, as well as the lack of COX-2 expression in HCT-116 cells [26]. This was further confirmed by Western blot analysis (Fig. 1b). Cellular uptake of [^18^F]Pyricoxib was significantly higher in HCA-7 cells compared to HCT-116 cells (Fig. 1c). After 90 min, uptake in HCA-7 cells reached 598 ± 93 % %radioactivity/mg of protein (*n* = 3), while uptake in HCT-116 cells was significantly lower reaching 317 ± 36 % %radioactivity/mg protein (*n* = 3) at the same time point.

COX-2 specificity of radiotracer uptake in HCA-7 cells was tested with cellular uptake inhibition studies using various selective COX-2 inhibitors. Cells were pre-incubated with 10 and 100 μM of various COX-2 inhibitors (celecoxib, rofecoxib, SC58125, and Pyricoxib) for 30 min prior to the addition of radiotracer [^18^F]Pyricoxib. Figure 1d shows inhibitory effects of selective COX-2 inhibitors at high concentration (100 μM) on the uptake of [^18^F]Pyricoxib normalized as %radioactivity/mg protein. The results in Fig. 1e demonstrate that radiotracer uptake in HCA-7 cells could be reduced in a concentration-dependent manner, although to a different extent.

Inhibition of radiotracer uptake was strongest with celecoxib which resulted in an inhibition of 35 % at 10 μM and 65 % at 100 μM, followed by Pyricoxib > rofecoxib > SC58125, respectively. Novel COX-2 inhibitor Pyricoxib inhibited radiotracer uptake by 35 and 50 % at inhibitor concentrations of 10 and 100 μM, respectively.

### Radiometabolite analysis

Radiometabolite analysis of blood samples revealed that radiotracer [^18^F]Pyricoxib was only slowly metabolized in NIH-III mice. The percentage of intact [^18^F]Pyricoxib decreased from 98 % at 5 min p.i. to 60 % at 2 h p.i. The content of detectable radioactivity in plasma fraction increased from 30 % after 5 min to 50 % after 2 h p.i., while radioactivity amount in the blood cell fraction decreased over time. The levels of radioactivity bound to plasma proteins did not change over 2 h and remained low in the range of 2 to 7 %. The following blood compartment distribution of radioactivity was determined—5 min: 68 % blood cells, 2 % plasma proteins, and 30 % plasma supernatant; 30 min: 54 % blood cells, 4 % plasma proteins, and 42 % plasma supernatant; and 60 min: 43 % blood cells, 7 % plasma proteins, and 50 % plasma supernatant, respectively.

### Dynamic PET imaging of HCA-7 tumor-bearing mice

**Fig. 2:**
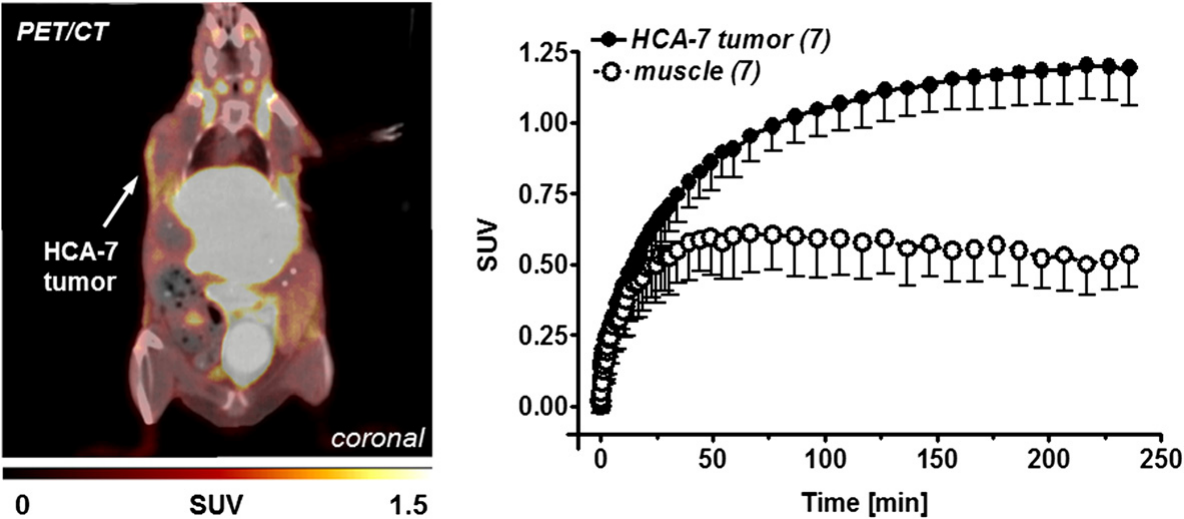
*Left*: PET/CT image (coronal slice) 2 h after injection of [^18^F]Pyricoxib into a HCA-7 tumor-bearing NIH-III mouse. 3.5 % HSA was added as carrier protein to the final injection solution. *Right*: Time-activity curves for tumor uptake of [^18^F]Pyricoxib and its clearance from muscle tissue over 4 h post injection. Data are shown as mean ± SD from seven dynamic PET experiments

Figure 2 shows a representative PET/CT image of a HCA-7 tumor-bearing mouse after 2 h p.i. of [^18^F]Pyricoxib. Analyzed time-activity curves (TACs) describe continuous increase of radioactivity accumulation in the tumor over 4 h reaching a standardized uptake value (SUV_4h_) of 1.19 ± 0.13 (*n* = 7). Radioactivity in the muscle as reference tissue peaked at 60 min p.i. (SUV_60min_ 0.61 ± 0.14) followed by a slow washout over time (SUV_4h_ 0.53 ± 0.11; *n* = 7) leading to a tumor-to-muscle ratio of 2.25 after 4 h p.i.

**Fig. 3:**
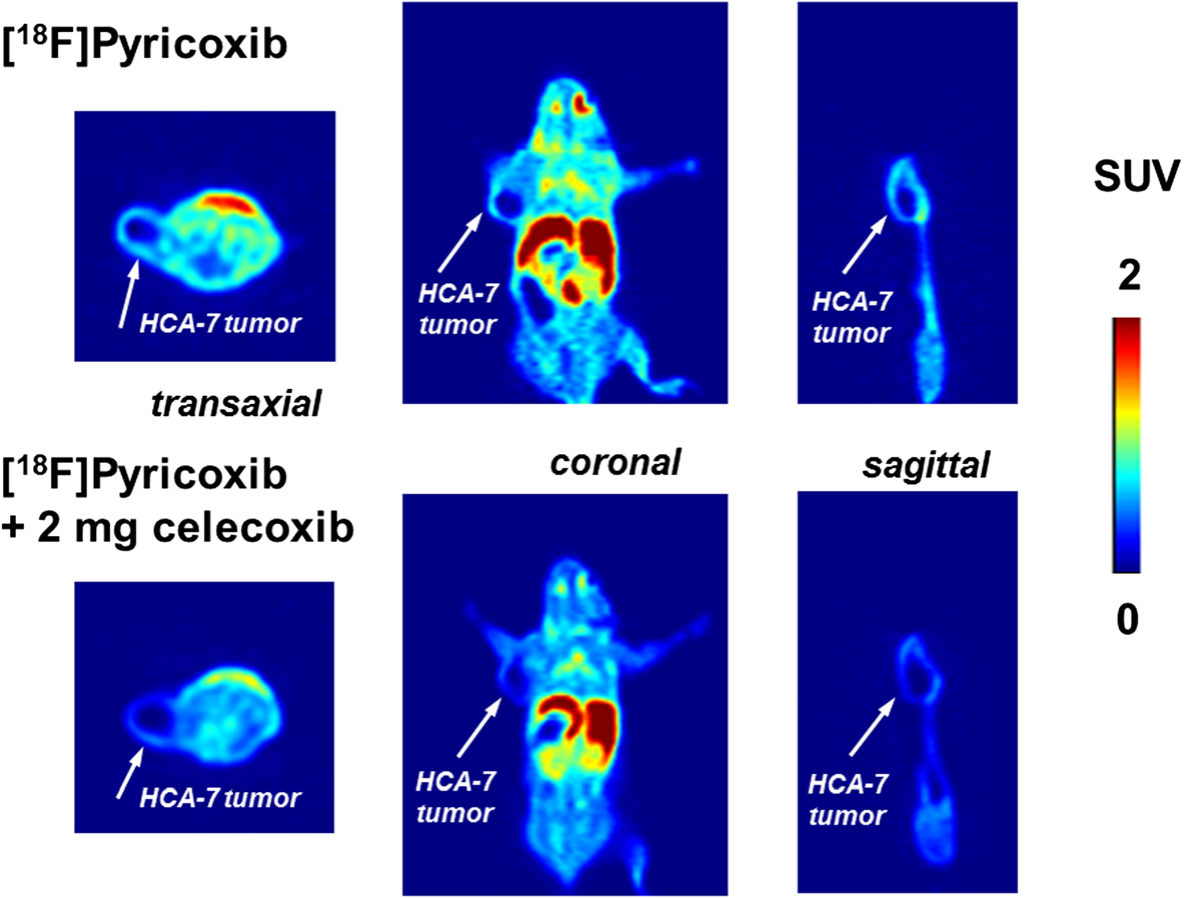
*Top*: transaxial, coronal, and sagittal PET images at 60 min p.i. of [^18^F]Pyricoxib into HCA-7 tumor-bearing NIH-III mouse (control); *bottom*: transaxial, coronal, and sagittal PET images at 60 min p.i. of [^18^F]Pyricoxib into HCA-7 tumor-bearing NIH-III mouse (pre-treated with 2 mg of celecoxib 60 min prior to radiotracer administration; no HSA added)

COX-2 specificity of radiotracer uptake in HCA-7 tumors was studied in a second series of experiments. Figure 3 depicts transaxial, coronal, and sagittal PET images of radioactivity distribution after 60 min p.i. in HCA-7 tumor-bearing mice without celecoxib (top) and in the presence of 2 mg of celecoxib given by i.p. injection 60 min prior to radiotracer administration.

**Fig. 4:**
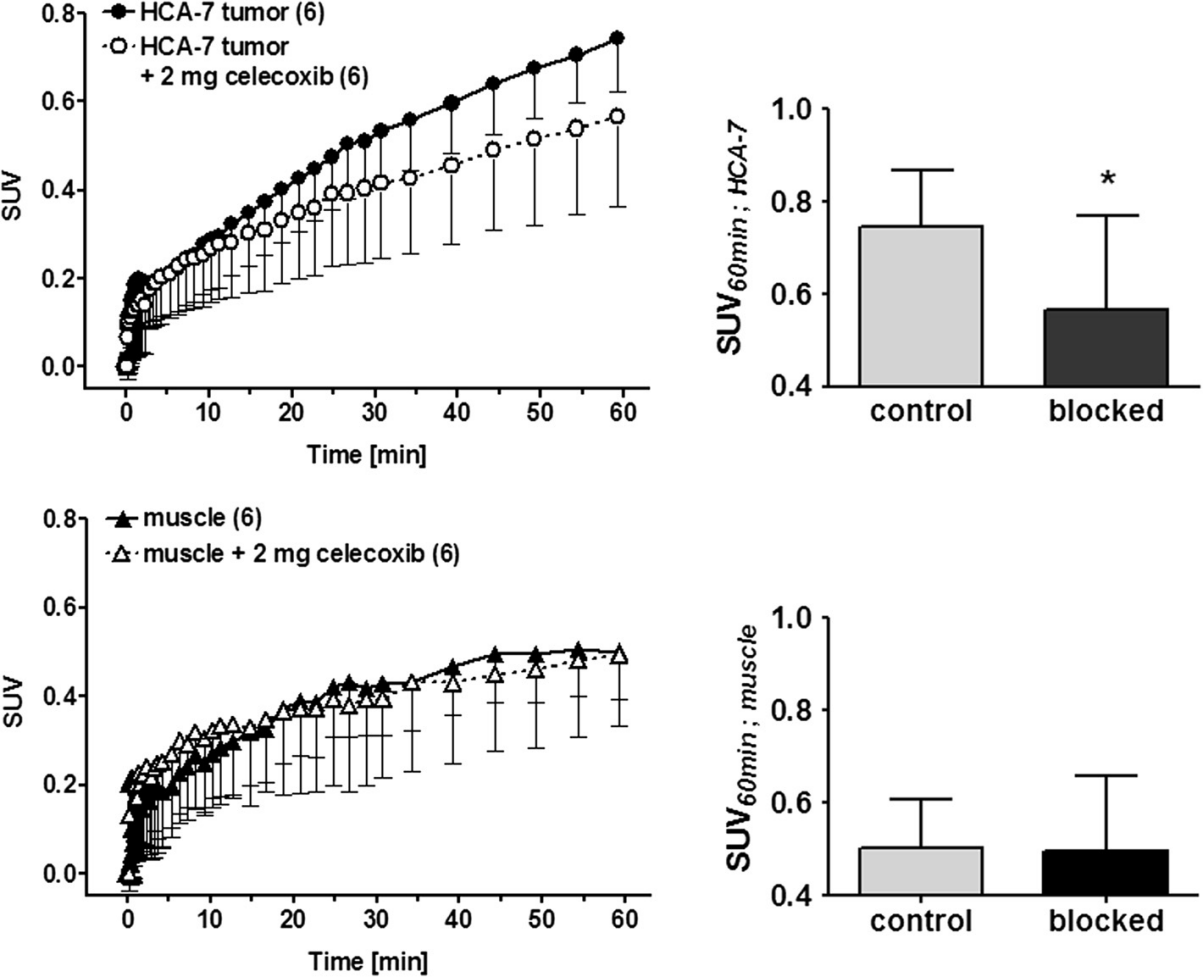
*Left*: analysis of PET imaging-derived standardized uptake values (SUV) for HCA-7 tumor uptake and muscle clearance over 60 min p.i. of [^18^F]Pyricoxib in the presence and absence of 100 mg/kg (2 mg per mouse) celecoxib. No HSA was added. *Right*: statistical analysis of the blocking effect with 2 mg celecoxib on the SUV at 60 min p.i. All data are shown as mean ± SD from six experiments. *P < 0.05

Tumors in the pre-treated animals clearly showed reduced radioactivity accumulation. Figure 4 summarizes quantification of blocking experiments. Each blocking experiment was performed on two consecutive days using the same animal first studied with a baseline scan (control) followed by a PET scan after pre-treatment the animal with celecoxib (2 mg).

Uptake of radiotracer [^18^F]Pyricoxib in HCA-7 tumors after 60 min p.i. in control animals resulted in a mean SUV of 0.75 ± 0.12 (*n* = 6), which was significantly higher than radioactivity uptake in pre-treated animals (SUV = 0.57 ± 0.20, *n* = 6; *P = 0.05).

SUV of 0.57 ± 0.20 in HCA-7 tumors of pre-treated animals was in the same range as the SUV measured in the muscle as reference tissue (0.50 ± 0.11; *n* = 6) at the same time point. Pre-treatment with celecoxib did not impair muscle uptake which is further indicative that observed inhibitory effects in tumor tissue were COX-2 mediated.

### Biodistribution studies in HCA-7 tumor-bearing mice

Results obtained from PET imaging experiments were confirmed by complementary biodistribution studies. Results of biodistribution experiments are summarized in Table 1. Pre-treatment with 2 mg of celecoxib (100 mg/kg) led to a significant reduction of radiotracer uptake into HCA-7 tumors. Control animals showed a tumor uptake of 2.12 ± 0.53 %ID/g after 60 min p.i., whereas celecoxib-treated animals displayed a significantly reduced tumor uptake of 1.04 ± 0.30 %ID/g (**P < 0.01).

**Table 1:** Biodistribution of [^18^F]Pyricoxib in control (left) and treated (right) HCA-7 tumor-bearing NIH-III mice (*n* = 4)

	[^18^F]Pyricoxib	[^18^F]Pyricoxib *+* 2 mg celecoxib
Organ		
Blood	0.65 ± 0.12	0.98 ± 0.09
Heart	2.52 ± 0.54	5.70 ± 1.58
Lung	3.75 ± 0.66	52.26 ± 43.08
Liver	17.13 ± 1.22	25.47 ± 2.44
Kidneys	4.38 ± 1.01	8.40 ± 0.77
Spleen	1.24 ± 0.33	2.72 ± 0.65
Stomach	1.08 ± 0.29	3.96 ± 2.73
Duodenum	4.59 ± 0.73	5.18 ± 1.31
Intestine (small)	8.08 ± 2.33	6.66 ± 1.95
Intestine (large)	4.21 ± 1.74	3.48 ± 0.80
Pancreas	3.92 ± 1.39	7.22 ± 2.87
Bone	0.67 ± 0.19	1.05 ± 0.16
Ovaries	8.22 ± 1.27	4.97 ± 2.84
Brain	1.74 ± 0.51	3.79 ± 0.56
Fat	12.67 ± 5.45	5.80 ± 3.84
Muscle	1.42 ± 0.54	1.59 ± 0.64
HCA-7 tumor	2.12 ± 0.53	1.04 ± 0.30**
Tumor/muscle	1.81 ± 1.04	0.70 ± 0.19
Tumor/blood	3.39 ± 0.55	1.09 ± 0.41

Data are displayed as means ± SD %ID/g after 60 min p.i

**P < 0.05

The presence of celecoxib also reduced uptake in fatty tissue, led to an increase in blood retention (**P < 0.01) as well as an increase in uptake in a number of other tissues and organs (the heart, lung, liver, kidney, stomach, pancreas, and brain). Increase in lung uptake confirmed the observed “first pass pulmonary retention” as a known phenomenon for selected secondary amines such as local anesthetics, e.g., lidocaine [27].

### PET imaging and biodistribution in HCT-116 tumor-bearing mice

**Fig. 5:**
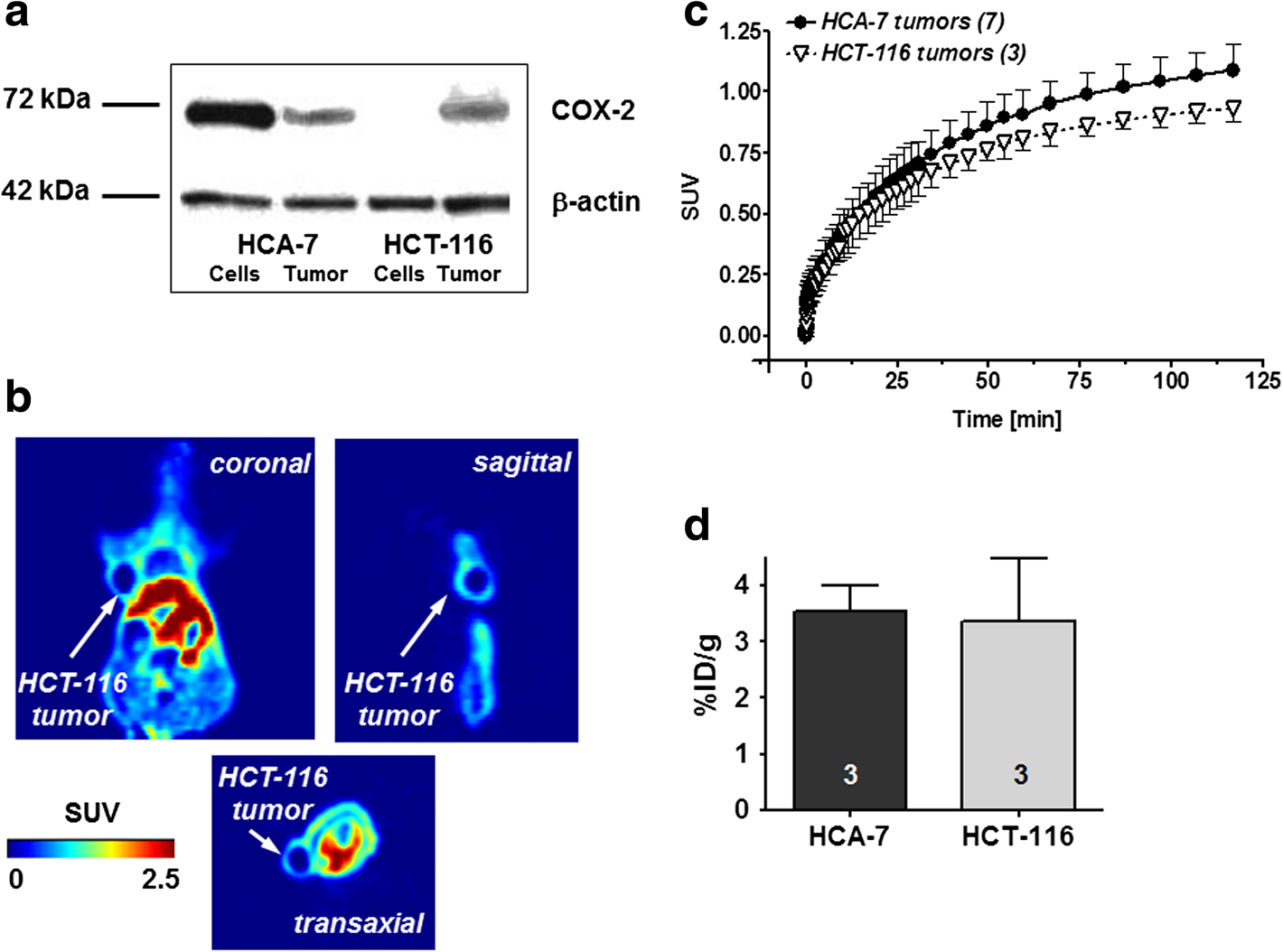
**a** Western blot analysis of COX-2 in HCA-7 and HCT-116 cell lysates as well as tumor samples generated from these cells. **b** Transaxial, coronal, and sagittal PET images at 120 min post injection of [^18^F]Pyricoxib into HCT-116 tumor-bearing NIH-III mouse. **c** Time-activity curves for HCT-116 tumor uptake of [^18^F]Pyricoxib in comparison to its uptake into HCA-7 tumors over 2 h post injection. Data are shown as mean ± SD from three dynamic PET experiments. **d** HCA-7 and HCT-116 tumor uptake determined from ex vivo biodistribution 2 h after injection of [^18^F]Pyricoxib. Data are shown as mean ± SD from three experiments each

In addition to the COX-2 expressing tumor cell line HCA-7, tumors were also generated from non-COX-2 expressing HCT-116 cells (see Fig. 1b). Injection of [^18^F]Pyricoxib into HCT-116 tumor-bearing mice resulted in comparable levels of radioactivity uptake as observed in HCA-7 tumors (Fig. 5b, c): SUV_2h_ 0.93 ± 0.06 (HCT-116; *n* = 3) versus 1.09 ± 0.13 (HCA-7; *n* = 3). Biodistribution analysis confirmed that finding (Fig. 5d). Analysis of COX-2 protein content in HCT-116 tumors confirmed COX-2 expression, which was absent in HCT-116 cells (Fig. 5a).

Induced expression of COX-2 upon injection of HCT-116 cells into mice explains the observed uptake of radiotracer [^18^F]Pyricoxib in HCT-116 tumors. This finding excludes HCT-116 tumors as a COX-2-negative tumor model in vivo.

### Immunohistochemical detection of COX-2 and CD68

**Fig. 6:**
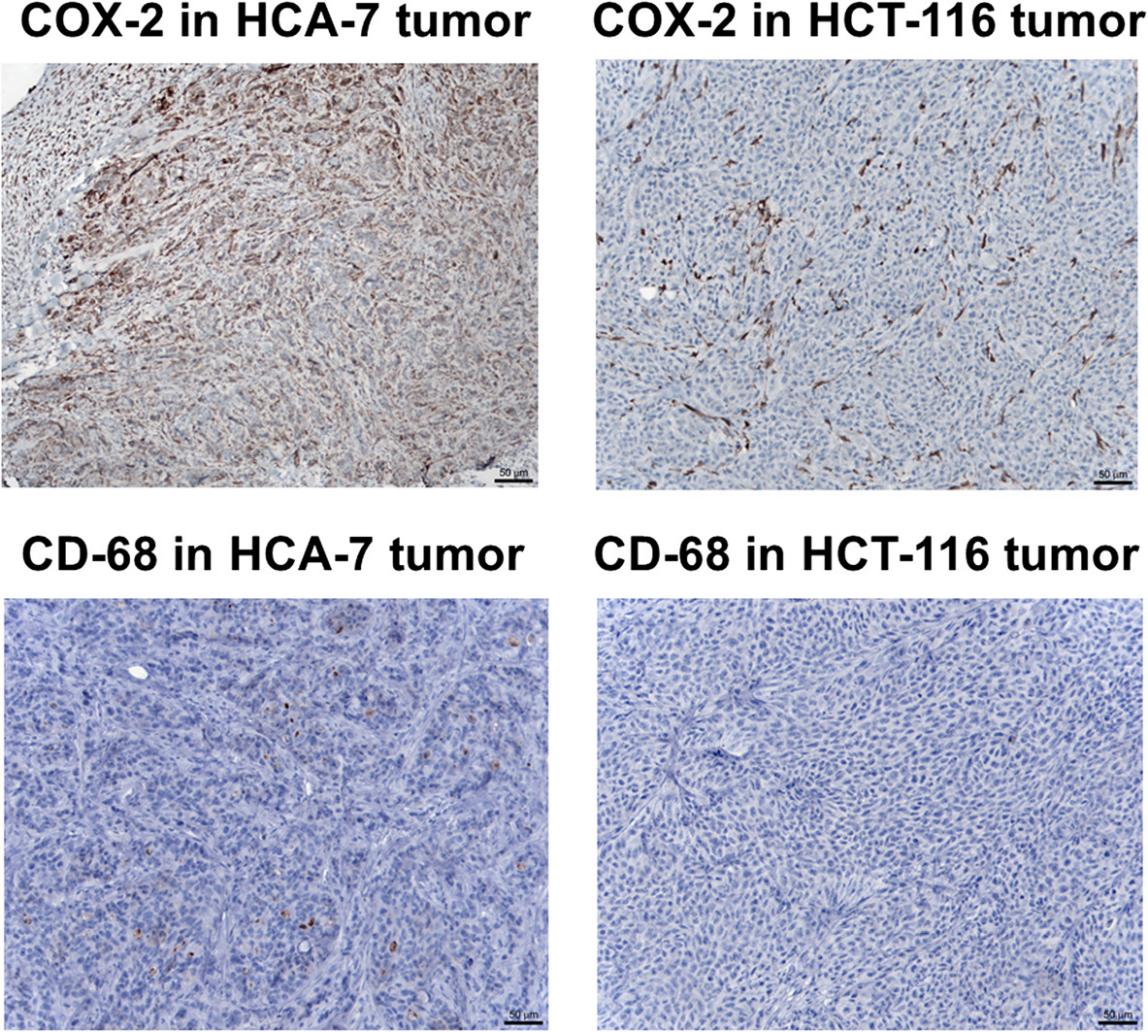
Immunohistochemical staining with COX-2 (*top*) and CD-68 (*bottom*) antibody in tumor tissue slices from NIH-III mice bearing HCA-7 (*left*) and HCT-116 (*right*) tumors

Immunohistochemical staining was used as an additional method to detect COX-2 protein in HCA-7 and HCT116 tumor tissue. In addition, tissues were also analyzed for CD68, which is a surface marker for various cells with macrophage/monocyte origin in association with inflammation processes [28]. As images in Fig. 6 show, a strong and clear staining for COX-2 was observed in HCA-7 tumor tissues.

In contrast, HCT-116 tumor tissue was mainly COX-2 negative, except of several inclusion cells, revealing very strong COX-2 staining intensity leading to some COX-2 expression in HCT-116 tissue samples. These inclusion cells morphologically may not represent tumor cells, but this has not been confirmed in full detail.

In addition, only very few CD68 positive cells were detected in HCA-7 tumors, while no staining for CD68 was detected in HCT-116 tumors.

## Discussion

Over the last two decades, numerous PET and SPECT radiotracers have been developed for COX-2 imaging. Several radiotracers have been subject of pre-clinical evaluation for molecular imaging of COX-2 expression in inflammation and cancer [17–19]. However, most of the reported radiotracers failed to visualize COX-2 in vivo due to multiple challenges including low metabolic stability, insufficient inhibitory potency and specificity for COX-2, and high non-specific binding to other targets. These challenges are especially pronounced for experiments aimed at molecular imaging of COX-2 in various pre-clinical cancer models. As a result, none of the reported radiotracers could demonstrate specific interaction with COX-2 in vivo. Therefore, to date, no successful imaging strategy for COX-2 in cancer has been described. The reasons for the failure of successful molecular imaging of COX-2 in vivo can be attributed to a major extent to unfavorable radiopharmacological profile of the radiotracers tested, but the selection and utilization of suitable pre-clinical models to study COX-2 radiotracers seems to be equally challenging.

In this study, we evaluated novel COX-2 radiotracer [^18^F]Pyricoxib in HCA-7 mouse xenografts as a pre-clinical model of colorectal cancer. Radiotracer [^18^F]Pyricoxib contains a 6-membered trifluoromethyl pyrimidine core structure and a methylsulfone COX-2 pharmacophore, which is different to most of the recently developed and tested COX-2 inhibitors like celecoxib and valdecoxib containing 5-membered heterocyclic core structure and a sulphonamide COX-2 pharmacophore [17–22].

Radiotracer [^18^F]Pyricoxib was prepared in good radiochemical yields of about 27 % using 4-[^18^F]fluorobenzylamine as readily available ^18^F building block [25] within a substitution reaction using 2-(methyl-sulfonyl)-4-(4-(methyl-sulfonyl)phenyl)-6-(trifluoro-methyl)-pyrimidine **1** as the radiolabeling precursor.

The radiotracer was shown to possess reasonable metabolic stability in vivo, reaching 60 % of intact [^18^F]Pyricoxib after 2 h p.i. in mice. Moreover, radiotracer [^18^F]Pyricoxib exhibited high inhibitory potency and selectivity for COX-2 (IC_50_ 7 nM) versus COX-1 (IC_50_ >100 μM).

IC_50_ values of various selective COX-2 inhibitors towards COX-2 inhibition such as celecoxib (40 nM, determined in the same assay as Pyricoxib [24]), SC58125 (10 nM [29]), and rofecoxib (18–26 nM [30]) confirm that Pyricoxib displays COX-2 inhibitory potency comparable or higher to that of selected COX-2 inhibitors used as blocking agents in this study. Based on its pharmacological properties, [^18^F]Pyricoxib meets two basic requirements for a successful PET imaging agents—high metabolic stability and high inhibitory potency and selectivity for the target in the nanomolar range. Data on muscle clearance profile of [^18^F]Pyricoxib as reference for non-target tissue clearance suggest that an optimal imaging time window for [^18^F]Pyricoxib would be at 120 min p.i. or longer rather than 60 min p.i.

However, COX-2 is located inside the membrane of the endoplasmic reticulum. Consequently, radiotracers need to cross various biological membranes to reach the COX-2 binding site. For this purpose, a favorable lipophilicity profile is required, and the lipophilicity of [^18^F]Pyricoxib was determined to be a log *P* of 3.37. This value is in the range to allow for passive diffusion, and it is also in the same range as reported lipophilicity values of other radiolabeled COX-2 inhibitors [17–19]. Cellular uptake studies of [^18^F]Pyricoxib in human colorectal cell lines HCA-7 and HCT-116 demonstrated significantly higher radiotracer uptake and retention in COX-2-positive HCA-7 cells. However, overall uptake of the radiotracer was also sufficiently high in COX-2-negative HCT-116 cells. This finding is indicative of a favorable passive diffusion profile of the radiotracer in combination with COX-2-mediated uptake and retention mechanisms in the case of COX-2-expressing HCA-7 cells. In COX-2-negative HCT-116 cells, several COX-2 independent uptake and retention mechanisms are likely to be responsible for the observed radiotracer uptake.

Immunohistochemical analysis confirmed a high expression of COX-2 in HCA-7 tumors with lower but noticeable COX-2 expression in HCT-116 tumors. This is consistent with the Western blot analysis of both HCA-7 and HCT-116 tumor samples (Fig. 5). Both tumors showed negative staining for CD68 as marker for tumor-associated macrophages [31]. This finding indicates that observed positive staining for COX-2 in HCT-116 tumors is not related to the infiltration of COX-2-expressing macrophages as an inflammatory response to tumor cell inoculation and tumor growth.

Reduction of radiotracer uptake in HCA-7 cells in response to pre-treatment with various COX-2 inhibitors in a concentration-dependent manner indicated that cellular uptake and retention of [^18^F]Pyricoxib is largely related to specific binding to COX-2.

However, blocking efficacy varied among the applied COX-2 inhibitors and was most evident with celecoxib. No complete blockage of radiotracer uptake could be achieved, and the remaining radioactivity levels of >35 % even at high inhibitor concentrations of 100 μM is indicative of some non-specific intracellular binding of the radiotracer.

Interactions of radiotracer [^18^F]Pyricoxib with COX-2 and non-COX targets would explain the observed broad variety in blocking efficacy using different COX-2 inhibitors, assuming that every used compound possesses a distinct affinity and selectivity profile for both COX and non-COX targets.

However, given the data determined during the present study, it is not possible to speculate about the nature of potential non-COX targets, although some secondary targets have been identified in the literature. Most of the research in this area focused on celecoxib [32–34]. COX-2 inhibitors like celecoxib do not interact with COX-2 alone; they can also interact with a variety of other molecular targets.

Celecoxib was shown to directly target Ca^2+^ ATPase, protein-dependent kinase 1 (PDK-1), cycline-dependent kinases (CDKs) in concert with various cyclins, and carbonic anhydrases (CA) [32]. Direct inhibition of these proteins by celecoxib allows the drug to exert anti-carcinogenic properties in a COX-2 independent manner. Although coxibs are selective for COX-2 over COX-1, the assumption that these molecules are truly “selective drugs” is faulty. The therapeutic efficacy of drugs like celecoxib and rofecoxib can not only be attributed to the inhibition of arachidonic acid metabolism through inhibition of COX-2 enzyme exclusively. Data on the non-COX affinity profile for Pyricoxib is not available, but since celecoxib, rofecoxib, and Pyricoxib share a number of key COX-2 pharmacophores, the possibility that they share a number of non-COX molecular targets must be considered. However, at this point, potential non-COX interactions of radiotracer [^18^F]Pyricoxib were not further analyzed during this study.

Pre-clinical PET imaging experiments provided further evidence of COX-2-mediated uptake of [^18^F]Pyricoxib in COX-2-expressing HCA-7 tumors.

Consistent with cellular uptake results in COX-2-expressing HCA-7 cells, this radiotracer showed steady uptake in HCA-7 tumors with no wash-out of radioactivity over time. COX-2-mediated retention of [^18^F]Pyricoxib in HCA-7 tumors was confirmed by in vivo blocking experiments.

Pre-dosing of HCA-7 tumor-bearing mice with 2 mg of celecoxib per mouse resulted in a 16 % decrease of radioactivity uptake in the tumor at 60 min p.i., while the remaining ~80 % may be related to non-specific and non-COX-2-mediated interactions. The biodistribution data in control and treated animals revealed a 50 % blocking effect which confirmed selective COX-2-mediated uptake of [^18^F]Pyricoxib in HCA-7 tumors.

However, overall uptake in the muscle was also high, and only very slow clearance of radioactivity from muscle tissue was observed. Under normal physiological conditions, muscle tissue does not express COX-2 [2].

Therefore, the observed high uptake and retention of radioactivity in muscle tissue may also be related to non-COX-2-mediated interactions of [^18^F]Pyricoxib. The literature provides some examples of possible mechanisms. A recent study showed that celecoxib is able to inhibit G-protein-coupled drug efflux pumps and thereby enhance the intracellular retention of drugs [32].

Drug efflux pumps serve as one possible example of targets for non-COX-2 specific interactions of molecules designed on the typical coxib structural scaffold.

A number of these non-COX targets have been identified for celecoxib as typical example of the coxib drug family [32–34]. A COX-2 radiotracer recently developed by Uddin et al. serves as a good comparison to [^18^F]Pyricoxib in terms of non-COX-2 specific interaction [21]. The researchers developed an ^18^F-labeled celecoxib derivative and evaluated the radiotracer in a COX-2 inflammation model and a COX-2 tumor model. They showed a reduction of radiotracer uptake in a carrageenan-induced inflammation model in response to pre-treatment with celecoxib. However, the observed overall uptake level of the radiotracer (SUV 0.2) might be too low to be COX-2 specific, especially considering the high non-specific uptake of celecoxib in a variety of organs and tissues. Celecoxib contains the typical sulfonamide COX-2 pharmacophore. Various sulfonamides including celecoxib are also known to have a low nanomolar affinity for the members of the carbonic anhydrase (CA) enzyme family [33].

A reduction in uptake of a radiolabeled celecoxib derivative in response to treatment with celecoxib might therefore be representative of an interaction with CA rather than with COX-2.

In contrast, [^18^F]Pyricoxib contains a methylsulfone COX-2 pharmacophore which does not interact with CAs, but [^18^F]Pyricoxib displayed COX-2 specific interactions in vitro and in vivo. However, the observed multiple non-COX-2-mediated interactions of radiotracer [^18^F]Pyricoxib still represent a major challenge.

However, it must be concluded that non-specific interactions of COX-2 inhibitors like [^18^F]Pyricoxib are inevitable due to their rather high lipophilic nature, which is necessary to cross biological membranes to reach the binding site of COX-2 located inside of the endoplasmic reticulum.

## Conclusions

We developed a novel PET imaging assay for non-invasive detection of functional expression of COX-2 in cancer using radiolabeled COX-2 inhibitor [^18^F]Pyricoxib. Despite the inherent major challenges associated with the development of COX-2 selective radiotracers for PET imaging of COX-2 in vivo, we believe that radiotracer [^18^F]Pyricoxib based on a pyrimidine scaffold does show the best and most selective tumor uptake profile in COX-2-expressing tumors as analyzed in a pre-clinical in vivo model so far. Our own previous and unsuccessful attempts of developing a celecoxib-based ^18^F-radiotracer [22] for imaging COX-2 in vivo would support that alternative structure selection.

The novel imaging assay represents an important basis for translation into a “first-in-man” clinical study to assess COX-2 in vivo. Non-invasive imaging of COX-2 expression with [^18^F]Pyricoxib in cancer would be useful for assessing COX-2-mediated effects on chemoprevention and radiosensitization using COX-2 inhibitors as an emerging class of anti-cancer drugs, especially for colorectal cancer.

## Additional file

Additional file 1: PET imaging of cyclooxygenase-2 (COX-2) in a colorectal cancer model.

## Abbreviations

COX-2: cyclooxygenase-2
HSA: human serum albumin
MAP: maximum a priori
PET: positron emission tomography
ROI: region of interest
SEM: standard error of means
SD: standard deviation
SUV: standardized uptake value
TAC: time-activity curve
