# Supplementary material for: PET imaging of cyclooxygenase-2 (COX-2) in a pre-clinical colorectal cancer model
Source: EJNMMI Res. 2016 Apr 26;6:37. doi: 10.1186/s13550-016-0192-9 (PMC4844587; doi:10.1186/s13550-016-0192-9)
Supplement: Additional file 1: — PET imaging of cyclooxygenase-2 (COX-2) in a colorectal cancer model. [file 13550_2016_192_MOESM1_ESM.docx]

Additional file 1

**PET imaging of cyclooxygenase-2 (COX-2) in a colorectal cancer model**

*Ole Tietz, Melinda Wuest, Alison Marshall, Darryl Glubrecht, Ingrit Hamann,*

*Monica Wang, Cody Bergman, Jenilee D. Way, Frank Wuest*

**Materials and Methods**

**General**

All regents and solvents were obtained from Sigma-Aldrich, unless otherwise stated and used without further purification. Thin-layer chromatography (TLC) was performed on Merck silica gel F-254 aluminum plates, with visualization under UV light (254 nm). TLCs were developed in 1% MeOH/CH_2_Cl_2_ and analyzed using a BAS-5000 reader (Fuji Photo Film Co., LTD) and Adaptive Image Deconvolution Algorithm (AIDA) Image Analyzer v.450 software. High performance liquid chromatography (HPLC) purifications and analysis were performed using a Phenomenex LUNA® C18 column (100Å, 250x10mm, 10mm) on a Gilson 322 Pump module fitted with a 171 Diode Array and a radioactivity detector.

**Radiosynthesis of *N*-(4-[^18^F]fluorobenzyl)-4-[4-(methylsulfonyl)phenyl]-6-(trifluoro-methyl)pyrimidin-2-amine ([^18^F]Pyricoxib)**

To 6 mg of radiolabeling precursor **1** was added 4-[^18^F]fluorobenzylamine ([^18^F]FBA) in 1 ml of THF (typically 1 GBq of [^18^F]FBA) in a sealed vessel. The reaction vessel was heated at 140°C for 30 min. The mixture was diluted in 10 mL water and passed onto a SepPak® C18 cartridge, the cartridge was washed with 5 mL water and radiotracer [^18^F]Pyricoxib was eluted with 3 mL of CH_3_CN. The volume of the solvent was reduced on a rotary evaporator to prepare a 1 ml 70/30 CH_3_CN/H_2_O formulation for HPLC purification. [^18^F]Pyricoxib was purified using HPLC (HPLC conditions: isocratic 70/30 CH_3_CN/H_2_O; flow rate 3mL/min) and the product collected at retention time of 12.6 min. The solvent was evaporated using a rotary evaporator under vacuum at 30 °C.

Decay-corrected radiochemical yield was 27±11%, and specific activity exceeded 40 GBq/μmol. [^18^F]Pyricoxib was then dissolved in EtOH and diluted in Krebs buffer for *in vitro* studies (% of EtOH in final volume: <1%). For *in vivo* studies, 20 μL of [^18^F]Pyricoxib in EtOH was added to 180 μL of saline solution (10 % EtOH / saline solution).

**Cell uptake studies**

Cells were routinely cultivated in Dulbecco’s Modified Eagle Medium: Nutrient Mixture F-12 (DMEM/F-12, in house) medium, supplemented with 10% (v/v) heat-inactivated fetal bovine serum (FBS, Gibco 12483), penicillin-streptomycin (1%; Gibco 15140), 2-[4-(2-hydroxyethyl)piperazin-1-yl]ethanesulfonic acid (HEPES, 10mM; Gibco 15630), and L-glutamine (2 mM; Gibco 25030) at 37ºC and 5% CO_2_ in a humidified incubator. For the uptake studies, cells were seeded in 12-well plates at a density of 400,000 cells/mL and grown to 90-95% confluence.

**Preclinical PET imaging**

All animal experiments were carried out in accordance with the guidelines of the Canadian Council on Animal Care (CCAC) and approved by the local animal care committee (Cross Cancer Institute, University of Alberta).

A transmission scan for attenuation correction was not acquired. The radioactivity of the injection solution in a 0.5 mL syringe was determined with a dose calibrator (Atomlab^TM^ 300, Biodex Medical Systems, New York, U.S.A.). After the PET emission scan was started the radioactivity was injected with a delay of approximately 15 s. Data acquisition continued for 60 min in 3D list mode. The dynamic list mode data were sorted into sinograms with up to 54 time frames (10x2, 8x5, 6x10, 6x20, 8x60, 10x120, 6x300 s). The frames were reconstructed using maximum a posteriori (MAP) reconstruction modes. The pixel size was 0.085x0.085x0.12 cm, and the resolution in the center field of view was 1.8 mm. Correction for partial volume effects was not performed. The image files were further processed using the ROVER v2.0.51 software (ABX GmbH, Radeberg, Germany). Masks defining 3D regions of interest (ROI) were set and the ROIs were defined by thresholding.

ROIs covered all visible tumor mass of the subcutaneous tumors, and the thresholds were defined by 50% of the maximum radioactivity uptake level for HCA-7 tumor in each animal.

**Protein Analysis**

50 µg of protein was loaded and run for 120V for 1 hour on IDGel (IR121s). Transfer from the SDS-PAGE to membrane was carried out at 4 ºC overnight at 35V. The membrane was then washed in PBS once for 5 minutes before blocking in 5% skim milk + 0.1% Tween-20 + PBS for 1 hour at room temperature (rt). The membrane was washed once with PBST (PBS+0.1%Tween-20) for 5 minutes, followed by incubation with the COX-2 primary antibody (1:500; Santa Cruz sc-70879 mouse monoclonal) or COX-1 primary antibody and β-actin primary antibody (1:1000; Sigma A5060 rabbit monoclonal) for 1 hour at rt. After two more washes with PBST for 5 minutes the membrane was incubated with the COX-2 secondary antibody (1:1500; Santa Cruz sc-2005 goat anti mouse) or COX-1 secondary antibody and β-actin secondary antibody (1:10000; Sigma A0545 goat anti rabbit) for 1 hour at room temperature. The membrane was washed with PBST 4 times for 5 minutes each, followed by one wash with PBS for 5 minutes. The membrane was incubated with a 1:1 mixture of substrates from Pierce ECL Western Blotting Substrate (Thermo Scientific 32209) for 1 min before gently rinsing with water.
